# Dunglison’s and Bell’s Medical Libraries

**Published:** 1842-09

**Authors:** 


### DUNGLISON'S AND BELL'S MEDICAL LIBRARIES.

We regret to learn from an advertisement on the cover of the last number of the first named journal, that its publication ceases from the date, ending with the excellent treatise of the late Dr. Hope on the Diseases of the Heart and Great Vessels. The universal pecuniary embarrassment of the country is pleaded by the publishers as a reason, the receipts for some time not having justified its continuance. We sincerely wish that the intimation given by them of the resumption of the work at some future day, may be realised.

The Quarterly Library of Dr. Bell is now the only periodical of the kind existing among us. It sustains its well-earned reputation. The last number is a very valuable one, containing the several treatises of Gordon, Hey, Armstrong and Lee on the history, pathology and treatment of *Puerperal Fever and Crural Phlebitis*, with an introductory essay by Dr. Meigs of the Jefferson Medical College. Now that its contemporary has stopped, this should, and no doubt will receive an accession of subscribers. It is certainly one of the cheapest as well as most commodious forms of increasing a library.

